# Acknowledgement to Reviewers of *Antibiotics* in 2017

**DOI:** 10.3390/antibiotics7010007

**Published:** 2018-01-25

**Authors:** Antibiotics Editorial Office

**Affiliations:** MDPI AG, St. Alban-Anlage 66, 4052 Basel, Switzerland

Peer review is an essential part in the publication process, ensuring that *Antibiotics* maintains high quality standards for its published papers. In 2017, a total of 41 papers were published in the journal. Thanks to the cooperation of our reviewers, the median time to first decision was 24 days and the median time to publication was 54 days. The editors would like to express their sincere gratitude to the following reviewers for their time and dedication in 2017:
Aihara, MichikoMasuda, HisakoAlam, Mohammad AbrarMcFarland, Lynne V.Aldred, KatieMelman, ArtemAntonini, GiovanniMester, PatrickAoki, ShunsukeMiotla, PawelAung, Meiji SoeMiro, JordiAvent, MinyonMoore, MichaelBaughn, Anthony D.Morita, YujiBelenky, PeterMoynihan, PatrickBell, BrianMurphy, CormacBhadbhade, Mohan M.Naber, KurtBiernasiuk, AnnaNarsimhan, GanesanBoulekbache-Makhlouf, LilaNguyen, CynthiaBraissant, OlivierNitiss, JohnCaffrey, PatrickOughton, MatthewCampos Rosa, JoaquínPablo Salvador, JuanCaputo, Gregory A.Pancholi, VijayChang, Geng-RueiParker, Suzanne L.Charbon, GodefroidPaskaleva, ElenaChattoraj, DhrubaPatel, HardikkumarChodosh, JamesPathak, AshishClarke, Anthony J.Peczuh, MarkClarke, DavidPinto, TatianaColom, JoanPrzybylski, PiotrCostelloe, CéireRadcliff, Fiona JaneCranfield, CharlesReid, Christopher W.Cudic, PredragReilly, JacquiDavy, ChristinaReynolds, HannahDe Sutter, AnRogawski, Elizabeth T.Dixon, NicholasRoque, FátimaDomínguez Rebolledo, Álvaro EfrénRudenko, NataliiaDrlica, KarlSabatier, Jean-MarcDuane, SineadSapi, EvaEdgerton, MiraSarovich, DerekErb, WilliamSauvageau, DominicEsteve-Romero, JosepSchaumburg, FriederFerraz, RicardoSeo, Keun-SeokField, KenSheedy, ClaudiaFigueiral, Maria HelenaSmani, YounesFleming-Dutra, KatherineSmits, Wiep KlaasFurneri, Pio MariaStein, DanielGademann, KarlStiles, BradleyGarvey, MarySubbiah, MuruganGeronikaki, AthinaSuda, KatieGroundwater, Paul W.Sugawara, AkihiroHerzon, Seth B.Tanaka, HiromitsuHilbert, FriederikeTian, YanpingHolmes, MarkTipple, CraigHoye, SigurdTorres-Barceló, ClaraJain, PrashantUndheim, KjellJohnning, AnnaValiquette, LouisJoukhadar, ChristianVashee, SanjayKaguni, JonVeloo, AlidaKauffman, Anne Kathryn MarieViale, PierluigiKelley, William L.Vioque, AgustinKimber, MatthewVolkova, VictoriyaKinder, DavidWagemans, JeroenLacal, Juan CarlosWertz, Philip W.Laverty, GarryWhite, StephenLecky, Donna M.Willcox, Mark DuncanLeonard, AlanYamamoto, ShingoLi, Xian-ZhiZakrzewska-Czerwińska, JolantaLipman, JeffreyŻesławska, EwaLugg-Widger, FionaZhao, YanpingMaddox, CarolZitko, Jan

